# From bristle to brain: embryonic development of topographic projections from basiconic sensilla in the antennal nervous system of the locust *Schistocerca gregaria*

**DOI:** 10.1007/s00427-024-00716-2

**Published:** 2024-05-01

**Authors:** George Boyan, Erica Ehrhardt

**Affiliations:** 1 Graduate School of Systemic Neuroscience, Biocenter, Ludwig-Maximilians-Universität München, Grosshadernerstrasse 2, Martinsried, 82152 Planegg, Germany; 2 Institute of Zoology, AG Ito, Universität Zu Köln, Zülpicher Str. 47B, 50674 Cologne, Germany

**Keywords:** Locust, Antenna, Basiconic sensilla, Embryogenesis, Topographic projections

## Abstract

The antennal flagellum of the locust *S. gregaria* is an articulated structure bearing a spectrum of sensilla that responds to sensory stimuli. In this study, we focus on the basiconic-type bristles as a model for sensory system development in the antenna. At the end of embryogenesis, these bristles are found at fixed locations and then on only the most distal six articulations of the antenna. They are innervated by a dendrite from a sensory cell cluster in the underlying epithelium, with each cluster directing fused axons topographically to an antennal tract running to the brain. We employ confocal imaging and immunolabeling to (a) identify mitotically active sense organ precursors for sensory cell clusters in the most distal annuli of the early embryonic antenna; (b) observe the subsequent spatial appearance of their neuronal progeny; and (c) map the spatial and temporal organization of axon projections from such clusters into the antennal tracts. We show that early in embryogenesis, proliferative precursors are localized circumferentially within discrete epithelial domains of the flagellum. Progeny first appear distally at the antennal tip and then sequentially in a proximal direction so that sensory neuron populations are distributed in an age-dependent manner along the antenna. Autotracing reveals that axon fasciculation with a tract is also sequential and reflects the location and age of the cell cluster along the most distal annuli. Cell cluster location and bristle location are therefore represented topographically and temporally within the axon profile of the tract and its projection to the brain.

**Supplementary Information:**

The online version contains supplementary material available at 10.1007/s00427-024-00716-2.

## Introduction

Adaptive behavior requires that environmental information is transferred from a sensory system to central decision-making centers in a manner that conserves the identity of the stimulus. One neural mechanism identified as contributing to this is the principle of labeled lines whereby sensory structures and their central targets form a point-to-point conducting system (see Pereira and Alves 2011; Petersen 2017; Henley 2021). In insects, a key sensory organ for detecting environmental stimuli mediating courtship, feeding, and oviposition is the antenna (Gewecke 1970; Göpfert and Robert 2001a, Göpfert and Robert 2001b; Jarman 2014; Hansson et al. 1996; Todi et al. 2004), and information transfer to the brain of olfactory information such as host odor quality, aggregation pheromones, and sex pheromones has been shown to involve labeled lines that are highly conserved (Masson and Mustaparta 1990; Keesey and Hansson 2021).

The antennal flagellum of the locust *S. gregaria* is an articulated structure that on hatching at the end of embryogenesis comprises 11 segments or meristal annuli (terminology from Chapman 2002) numbered sequentially from distal to proximal along the antenna. Chapman (2002) showed that during subsequent postembryonic development, the more proximal annuli subdivide in a fixed manner to generate the adult complement of 24 annuli, but the most distal six annuli do not subdivide so their identity must already be established earlier during embryogenesis. These distal annuli bear a spectrum of sensilla that respond to various sensory stimuli (Hansson et al. 1996; Ochieng et al. 1998; Chapman 2002) and whose sensory neurons direct projections to the brain (see Hansson et al. 1996). Among these sensilla are the basiconic-type bristles (see Slifer et al. 1957, 1959) that have been implicated in olfaction, gustation, and mechanoreception (Hansson et al. 1996; Ochieng et al. 1998; Boronat-Garcia et al. 2022; Cassau et al. 2023). Strikingly, on hatching, basiconic-type sensilla are not only found at fixed locations on an annulus, but also only on the most distal six annular segments of the antenna that form during embryogenesis (Chapman 2002). This organization could therefore subserve information transfer to the brain along unmixed communication lines.

In our study here, we examine the developmental aspects of the sensory system involving these basiconic-type sensilla. We identify mitotically active sense organ precursors for sensory cell clusters in the epithelium of the early embryonic antenna; follow the subsequent appearance of these clusters and their neuronal progeny along successive annuli; and map the organization of axonal projections from such clusters into the antennal tracts running to the brain. Cell clusters first appear distally and then in a proximal direction within the epithelium so that sensory neuron populations are distributed in an age-dependent manner along the antenna. Axon fasciculation with a tract is also sequential and reflects the location and age of the cell cluster along the flagellum. Cell cluster location and hence sensilla location on the cuticle are therefore represented topographically and temporally within the axon profile of the afferent tract to the brain. We compare our findings with other studies which show how topographic information transfer contributes to brain maps of sensory perception.

## Materials and methods

### Animal source

Locusts (*Schistocerca gregaria*) were raised in crowded colonies at the Biocenter, Ludwig-Maximilians-Universität München, with a 12/12-h light/dark regime, 35% air humidity, a day temperature of 30 °C, a night temperature of 20 °C, and in continuously circulating air. Eggs were incubated in moist, aerated containers under this same regime. Embryos were staged to the nearest 1% of developmental time (5% ≈ 24 h under colony conditions) according to Bentley et al. (1979). The results presented in this paper derive from experiments on over 140 preparations.

### Immunolabeling

Protocols for immunolabeling with primary and secondary antibodies, microscopy, and image processing were all as previously described (see Boyan and Williams 2004, 2007; Boyan and Niederleitner 2011; Ehrhardt et al. 2015, 2016).

The following antibodies were employed:

#### Primary antibodies

Anti-horseradish peroxidase (α-HRP, polyclonal rabbit, Dianova 323–005-021) recognizes a cell surface epitope first reported as being specific to neurons in *Drosophila* and grasshopper embryos (Jan and Jan 1982). Labeling has subsequently also been observed on neuronal precursors, on neuron-like cells such as pioneers (Boyan and Ehrhardt 2017, 2020), and on developing non-neuronal epithelial cells (Caudy and Bentley 1986). In insect embryos, the HRP antibody binds to several proteins which bear the HRP epitope including neurotactin, fasciclin I, fasciclin II, neuroglian, and a receptor-linked protein tyrosine phosphatase (Desai et al. 1994). For double labeling with α-PH3, the primary HRP antibody was polyclonal in goat (Dianova, 123–005-021).

Anti-Lachesin (α-Lach, Mab 1C10, mouse, generous gift of M. Bastiani) recognizes the glycosylphosphatidylinositol (GPI)-linked cell surface molecule Lachesin belonging to the Ig superfamily (see Karlstrom et al. 1993). The expression occurs initially on all differentiating neuroepithelial cells, but only neurons and cells involved in neurogenesis, such as precursors, continue to express the molecule later.

#### Cell proliferation marker

Anti-phospho-histone H3 (α-PH3, Ser10, polyclonal rabbit, Millipore 06–570) recognizes and binds the phosphorylated form of the amine terminal of histone 3. This binding is only possible when the chromatin lies dissociated from the nucleosome complex, as occurs during mitotic chromosome condensation (see Hendzel et al. 1997; Adams et al. 2001 for details).

#### Lineage tracer

5-Ethynyl-2′-deoxyuridine (EdU) is a thymidine analog which is incorporated into the DNA of proliferating cells during the S-phase of the cell cycle (Sousa-Nunes et al. 2011; Takagi et al. 2012). Thereafter, EdU is retained by those cells deriving from such a mitotically active precursor. Columns of EdU-positive cells that are in direct cell–cell contact may be considered to represent a lineage within the epithelium (see Hsu 2015). A Click-iT® EdU imaging kit (Invitrogen, C10337) was used for EdU incorporation experiments. The protocol followed is as described in Boyan and Ehrhardt (2017).

#### Nuclear marker

DAPI (4,6-diamidino-2-phenylindole, Sigma) is a cell-permeable fluorescent probe which binds to the minor groove of double-stranded DNA (Naimski et al. 1980). The protocol followed is as described in Boyan and Ehrhardt (2017).

#### Secondary antibodies

In single labeling, the secondary against α-HRP was Alexa® 488 (goat anti-rabbit, Invitrogen) or Cy3 (goat anti-rabbit, Dianova); against α-Lachesin, it was Cy3 (goat anti-mouse, Dianova); against α-PH3, it was donkey anti-rabbit Cy3 (Dianova, 711–165-152). In double labeling involving α-PH3/α-Lachesin, the combinations were goat anti-rabbit Alexa® 488 (Invitrogen, A11034) against α-PH3 and goat anti-mouse (GAM-Cy3, Dianova) against α-Lachesin. In triple labeling involving α-HRP/α-Lach/α-PH3, the combination was Cy5 (donkey anti-goat, Dianova) against α-HRP, Alexa® 488 (donkey anti-mouse, Invitrogen) against α-Lachesin, and donkey anti-rabbit Cy3 (Dianova, 711–165-152) against α-PH3. False colors were applied to images to distinguish each label.

Controls for the specificity of all secondary antibodies were (a) the lack of a staining pattern in the absence of the primary antibody and, (b) in all cases, a staining pattern consistent with previously published data (see above).

### Embedding and sectioning

Embedding of antennae for sectioning was previously described in Boyan and Williams (2004) for agarose medium (embryos) and in Boyan and Williams (2007) for Epon 812 medium (nymphs). Preparations were routinely sectioned at 14-µm thickness.

### Imaging

#### Light microscopy

Cuticular bristles were viewed under a Zeiss Axioskop2 compound microscope using both transmission and differential interference contrast (DIC) optics. Images were captured with a 1.3-MP CCD camera (Scion Corp.) using Scion Visicapture™ software.

#### Confocal microscopy

Optical sections of preparations were acquired with a Leica TCS SP5 confocal laser scanning microscope with × 20 and × 63 oil immersion objectives. Fluorophores were visualized using excitation wavelengths of 405 nm for DAPI; 488 nm for Alexa® 488 and EdU; 561 nm for Cy3; and 633 nm for Cy5. All images were processed using public domain software (ImageJ). False colors were applied where necessary and adjustments were made to resolution, brightness, and contrast. Final figures were formatted using Canvas X™ software (ACD Systems).

## Nomenclature

Antennal axes are named with respect to the head capsule. The base of the antenna is considered proximal, and the tip of the antenna is considered distal. In addition, the outer epithelium of the antenna is considered being of ectodermal origin, and the inner lumen of the antenna is of mesodermal origin (Snodgrass 1935; Butt 1960; Kotrla and Goodman 1984; Kuwada and Goodman 1985; Boyan and Williams 2007).

## Results

### Cuticular bristles and their innervation

**Fig. 1:**
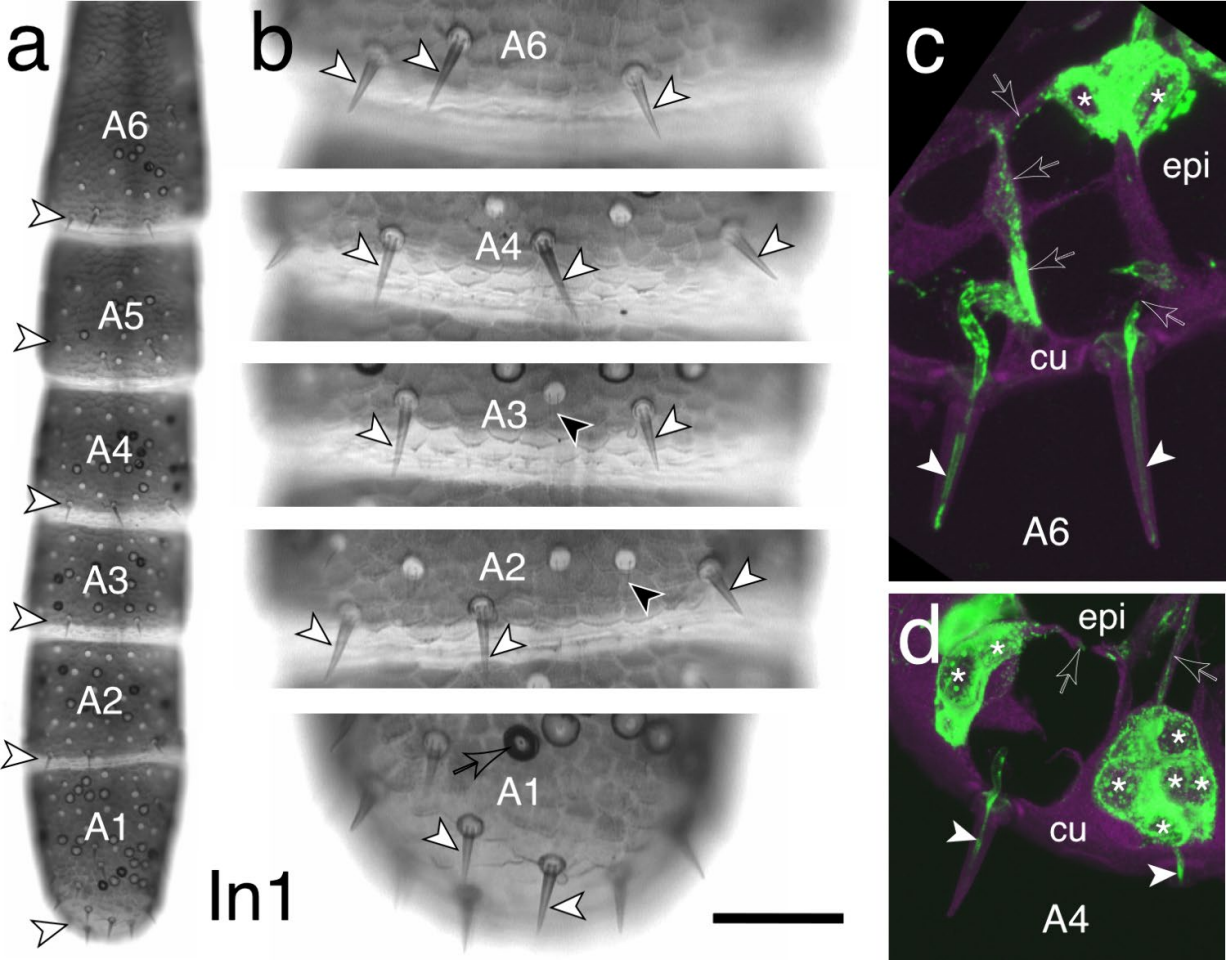
Cuticular bristles and their innervation on the antenna of a first instar (In1) locust. **a** Low power photomicrograph of the ventral cuticular surface of the six most distal segments or annuli (A6, A5, A4, A3, A2, A1; terminology from Chapman 2002) of the antenna. Prominent bristles (white arrowheads) are typically found near the distal border of each segment. Distal is to the bottom. **b** Photomicrograph at higher power shows the bristles in the regions indicated panel a (open white arrowheads) to be of the basiconic-type (see Suppl. Figure 1). According to Chapman (2002), these are only found on the six most distal annuli and have a consistent location in each. Other sensilla present in the images but not part of this study include coeloconic (open black arrowhead) and campaniform sensilla (black arrowheads) (for details of receptor morphologies, see Slifer et al. 1957, 1959; Chapman and Greenwood 1986; Ochieng et al. 1998; Chapman 2002). **c**, **d** Confocal images following neuron-specific α-HRP immunolabeling (green) show clusters of sensory cells (white stars) in the epidermis (epi) associated with basiconic-type bristles on the cuticle (cu) of annuli A6 (panel c) and A4 (panel d) in longitudinally sectioned antennae from a first instar locust. Cuticular and epidermal regions autofluoresce (false color magenta). A fasciculated bundle of HRP-positive neurites (open/white arrow) projects from each epidermal cell cluster and terminates as a dendrite (white arrowhead) at the tip within each bristle, consistent with the innervation of basiconic-type sensilla (see Suppl. Figure 1). Fasciculated axons (open white arrowhead) exit each cell cluster en route to the antennal nerve (not imaged here but see Fig. 6). Scale bar represents 180 µm in **a**, 45 µm in **b**, 15 µm in **c**, **d**

Light microscopic examination of the antenna from a 1st instar (In1) locust (Fig. 1a, b) reveals prominent bristles whose locations on the ventral cuticular surface of only the six most distal annuli (A6, A5, A4, A3, A2, A1) appear to be fixed, consistent with the findings of Ochieng et al. (1998) and Chapman (2002) for basiconic sensilla. Chapman (2002) reported further that on hatching, basiconic-type sensilla are found only on the most distal six annular segments of the antenna.

Immunolabeling against horseradish peroxidase (α-HRP, see Methods) in sectioned antennae at the In1 stage reveals clusters of HRP-positive sensory cells in the epidermis of distal annuli such as A6 (Fig. 1c) and A4 (Fig. 1d). Note that as these are sectioned antennae, the complete cell cluster comprising over ten cells is not imaged (see Figs. 4, 5). Each cluster is associated with a fasciculated HRP-positive neurite that terminates as a dendrite within the tip of the bristle, and an axon exits each cell cluster en route to the antennal nerve (not imaged here but see Fig. 5). The overall morphology and innervation pattern we report here are consistent with those of basiconic-type sensilla as previously described by Slifer et al. (1957, 1959; Suppl. Figure 1).

### Ontogeny and development of sensory cell lineages in apical segments of the embryonic antenna

**Fig. 2:**
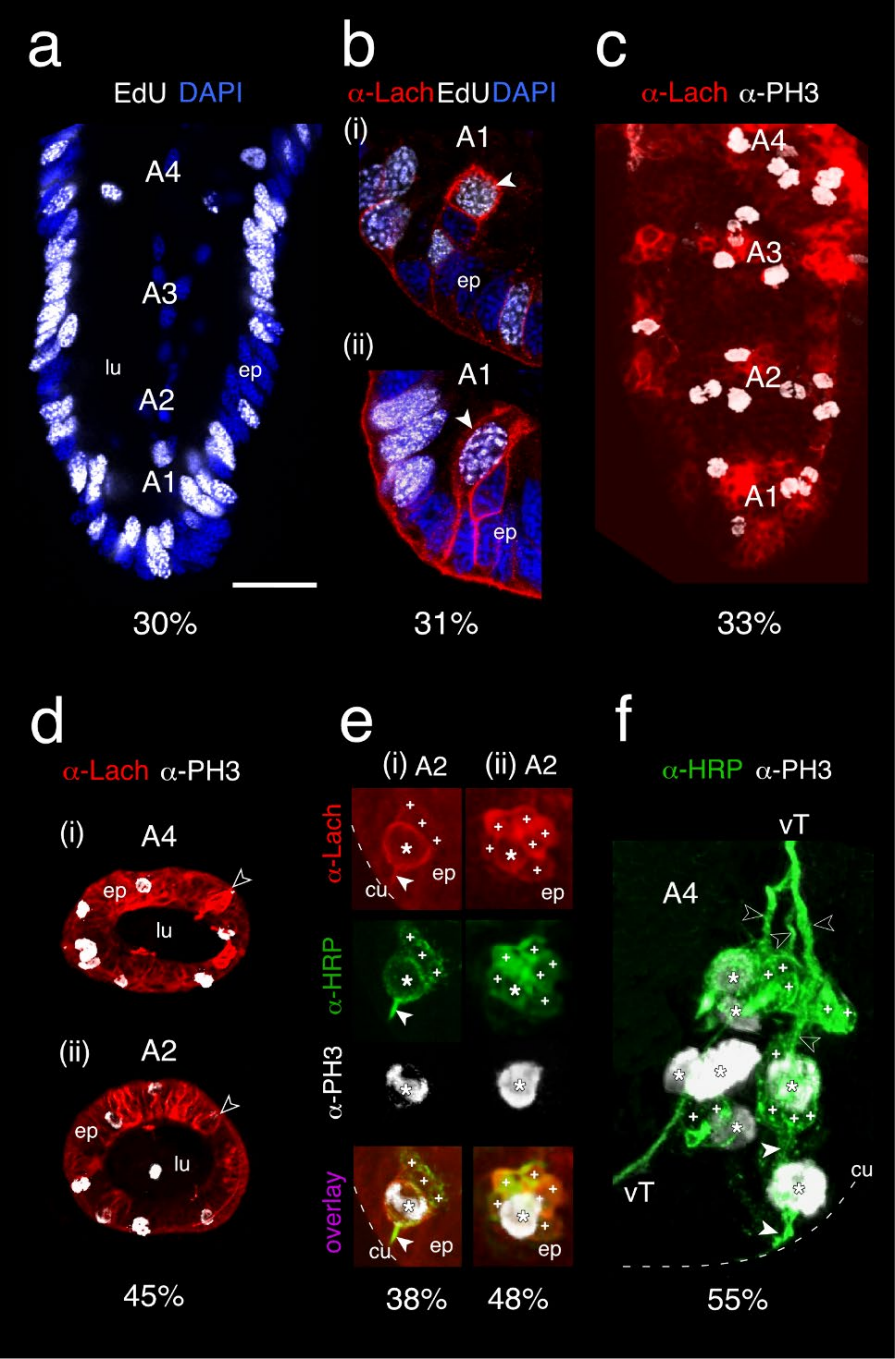
Ontogeny and development of sensory cell lineages in apical segments of the embryonic antenna. **a** Culturing with the S-phase label EdU (white) and co-labeling with the nuclear stain DAPI (blue) reveals differentiating SOPs in the epithelium (ep) of annuli A1, A2, A3, and A4 at 30% of embryogenesis. Unidentified differentiating cells are also present in the lumen (lu) of A1 and A4. Distal is to the bottom. **b** Triple labeling against Lachesin (α-Lach, red), against the S-phase label EdU (white), and using the nuclear stain DAPI (blue) shows an EdU-labeled, Lach-positive SOP (white arrowhead) differentiating at the tip of a column of epithelial (ep) cells in A1 of repeat preparations (i, ii) at 31% of embryogenesis. **c** Confocal image following double labeling against Lachesin (α-Lach, red) and the proliferative cell marker phospho-histone 3 (α-PH3, white) at 33% of embryogenesis reveals mitotically active precursor cells restricted to the Lach-positive epithelial domains of A1, A2, A3, and A4. **d** Confocal images of antennal annuli A4 (i) and A2 (ii) in cross-section at 45% of embryogenesis following double labeling against Lachesin (α-Lach, red) and anti-phospho-histone 3 (α-PH3, white). PH3-positive mitotically active precursors are located circumferentially in the epithelial domain (ep) of each annulus, as are Lach-positive differentiating cell clusters (open white arrowheads). A single mitotically active cell is located in the lumen (lu) of A2. Panel A4 modified from Boyan and Ehrhardt (2020). **e** Confocal images following triple labeling against Lachesin (α-Lach, red), horseradish peroxidase (α-HRP, green), and phospho-histone 3 (α-PH3, white) reveal SOPs and associated HRP-positive/Lach-positive cells in annulus A2 of the embryonic antenna. At 38% (i), a representative Lach-positive proliferative SOP (white star) is associated with three Lach-/HRP-positive cells (white crosses). The SOP is typically linked to the cuticle (cu, dashed white line) by an epithelial foot (white arrowhead; terminology follows Locke and Huie 1981). Modified from Boyan and Ehrhardt (2020). At 48% (ii), a mitotically active SOP (white star) has detached from the epithelial cell layer and is associated with at least six Lach-/HRP-positive cells (white crosses). **f** Confocal image following double labeling against horseradish peroxidase (α-HRP, green) and phospho-histone 3 (α-PH3, white) at 55% of embryogenesis reveals mitotically active SOPs (white stars) each associated with a cluster of HRP-positive cells (white crosses) in annulus A4 of the antenna. Axonal processes (open white arrowheads) project centrally onto the ventral tract (vT), one of the two axon tracts projecting proximally to the antennal base. Fused dendritic processes (white arrowheads) from HRP-positive cell clusters associated with SOPs (located behind the more peripheral SOP) project peripherally towards the cuticular edge of the antenna (cu, dashed white) where they may become associated with sensilla that develop later (see Suppl. Figure 2). Scale bar represents 25 µm in **a**, 18 µm in **b**, 25 µm in **c**, 60 µm in **d**, 20 µm in **e**, 30 µm in **f**

To identify sense organ precursors (SOPs) differentiating in the epithelium of apical annuli A1–A4 and then generating sensory cell clusters, we cultured early embryos with the S-phase label EdU (see Methods) and subsequently co-labeled with the nuclear stain DAPI (Fig. 2a). Confocal imaging at 30% of embryogenesis reveals that differentiating SOPs are localized to defined bands in the epithelium each corresponding to an annulus. At 31%, triple labeling against Lachesin (α-Lach, a marker for differentiating neuroepithelial cells), against the S-phase label EdU, and the nuclear stain DAPI reveals differentiating SOPs, each associated with their own column of EdU-positive epithelial cells that represent lineages in A1 (Fig. 2b i, ii; see Methods; Hsu 2015). Double labeling against α-Lach and the proliferative cell marker phospho-histone 3 (α-PH3) demonstrates that at 33% of embryogenesis (Fig. 2c), mitotically active SOPs are distributed circumferentially within the Lach-positive epithelial domains of these distal annuli (Fig. 2d).

Developing neuronal lineages associated with SOPs were identified by triple labeling against Lachesin (α-Lach), horseradish peroxidase (α-HRP), and phospho-histone 3 (α-PH3) (Fig. 2d). At 38%, a representative Lach-positive proliferative SOP in annulus A2 is associated with three Lach-/HRP-positive progeny (Fig. 2d, i). The SOP is typically connected to the cuticle by an epithelial foot (see Locke and Huie 1981). At 48% (Fig. 2d, ii), a typical mitotically active SOP is now detached from the epithelium and is associated with at least six Lach-/HRP-positive progeny. At 55% of embryogenesis (Fig. 2e), double labeling against neuron-specific horseradish peroxidase (α-HRP) and phospho-histone 3 (α-PH3) reveals mitotically active SOPs each associated with a cluster of HRP-positive progeny. These have now generated axonal processes that project onto an antennal tract running to the brain (see Boyan et al. 2023). Fused dendritic processes from each cell cluster project peripherally to putatively innervate later developing cuticular sensilla.

### Temporal appearance of sensory cell clusters

**Fig. 3:**
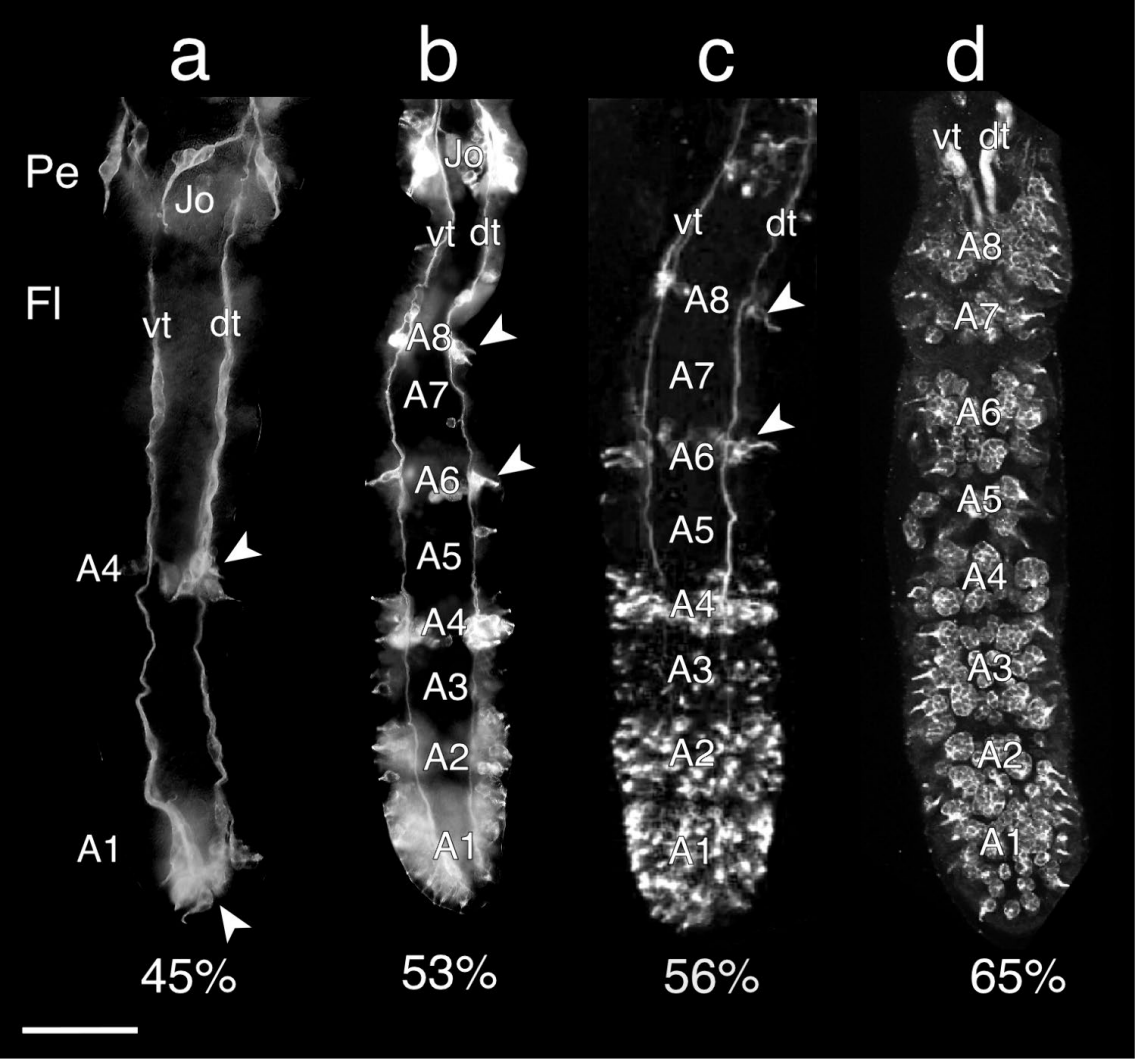
Immunolabeling against horseradish peroxidase (α-HRP) shows that sensory cell clusters appear in a distal to proximal direction along the antenna during embryogenesis. The arrow points to the antennal base. **a** At 45%, fluorescence photomicrograph shows sensory cell clusters (white arrowheads) in A1 of the flagellum (Fl), with a single cluster present unilaterally in A4. Neurons from these clusters project axons topographically onto either the ventral (vT) or dorsal (dT) antennal tracts running proximally to the antennal base. Cell clusters of Johnston’s organ (Jo) are also present in the pedicellum (Pe). **b** At 53%, fluorescence photomicrograph shows increasing numbers of cell clusters in A1, A2, and A4, while isolated sensory cell clusters are now evident in A6 and A8 (white arrowheads) along with their peripherally projecting fused dendrites. **c** At 56%, the confocal image reveals greater numbers of cell clusters are present throughout A1, A2, and A4 and also appear in A3. Note that cell cluster numbers in more proximal annuli A5–A8 are essentially similar to those of 53%. **d** At 65%, the confocal image shows cell cluster numbers have increased basally and now fill the epithelia of A1–A8. Panels a and b modified from Boyan and Williams (2004). Scale bar represents 90 µm in **a**, 165 µm in **b**–**d**

Immunolabeling against α-HRP reveals that labeled sensory cell clusters appear in a distal to proximal direction along the flagellum of the antenna during embryogenesis (Fig. 3). At 45% (Fig. 3a), significant numbers of HRP-labeled sensory cell clusters are only present in A1, with just an initial small cluster present unilaterally in A4. HRP-positive clusters belonging to Johnston’s organ are also visible in the pedicel. At 53% (Fig. 3b), more HRP-positive cell clusters appear in A1, A2, and A4 while isolated sensory cell clusters appear in A6 and A8. At 56% (Fig. 3c), greater numbers of HRP-positive cell clusters are present throughout A1, A2, and A4 and also appear in A3, but numbers in more proximal annuli A5–A8 have not advanced significantly compared to 53%. At 65% (Fig. 3d), labeled cell clusters now cover the epithelia of A1–A8. Proliferative precursors are no longer found in the epithelium of these distal annuli after this age (data not shown), suggesting that embryonic generation of clusters in annuli A1–A8 of the flagellum is complete.

We interpret these data as showing that the oldest neuronal population is found in cell clusters located distally near the antennal tip with progressively younger populations located more proximally towards the base.

### Topographic projections from cell clusters to antennal tracts

**Fig. 4:**
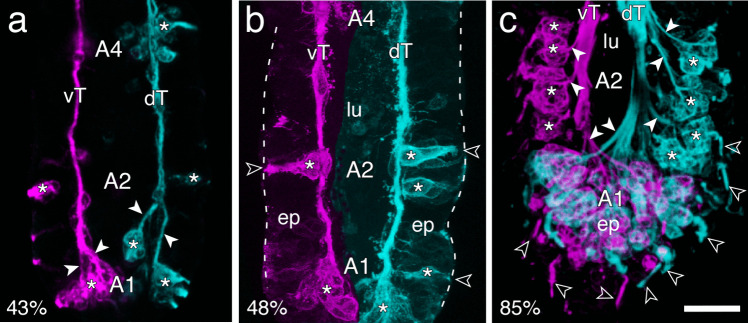
Developing patterns of fasciculation and topological organization of projections from sensory cell clusters to the two primary axon tracts that project proximally to the antennal base. Confocal images follow labeling against horseradish peroxidase (α-HRP). Ventral cell clusters and axon tracts are shown in false color magenta and dorsal cell clusters and tracts in false color cyan. Distal is to the bottom in all panels. **a** At 43% of embryogenesis, cell clusters (white stars) have appeared in distal segments A1, A2, and A4 of the antenna. Clusters in A1 have generated axons (white arrowheads) that project topologically onto the primary tract system (vT, dT) of the antenna. **b** At 48%, sensory cell clusters (white stars) arrayed in the epithelium (ep) are clearly associated with either a ventral (magenta) or dorsal (cyan) axon tract. Each tract runs along the border between the lumen (lu) and epithelium towards the antennal base. Dendrites (open white arrows) from some cell clusters can be seen to extend towards the cuticle (dashed white) but bristles are not yet evident. **c** Confocal image montaged from optical stacks collated according to depth demonstrates that at 85% of embryogenesis distal HRP-positive cell clusters (white stars label some) located ventrally (magenta) or dorsally (cyan) in the epithelium (ep) of A1 and A2 project axons (white arrowheads) topologically onto respective ventral (vT, magenta) or dorsal (dT, cyan) antennal tracts. Note the symmetrical projection patterns. Fused HRP-positive dendrites from these clusters innervate ventral (magenta) or dorsal (cyan) cuticular bristles (open white arrowheads). Scale bar represents 33 µm in **a**, 35 µm in **b**, 25 µm in **c**

We then investigated the development of projections from sensory cell clusters in the most distal annuli to the tract system of the antenna by labeling with α-HRP (Fig. 4). At 43% of embryogenesis (Fig. 4a), cell clusters have appeared in distal segments A1, A2, and A4 of the antenna and those in A1 have generated axons that project topologically onto the primary tract system. At 48% (Fig. 4b), sensory cell clusters arrayed in the epithelium are clearly associated with either a ventral or dorsal axon tract each running along the border with the lumen proximally towards the antennal base. Dendrites from some cell clusters can be seen to extend towards the cuticle but bristles are not yet evident. Even at 70% of embryogenesis (Suppl. Figure 2), sensory cell clusters arrayed in the epithelium project dendrites to the cuticle edge of the antenna, but target basiconic bristles are not yet evident. At 85% of embryogenesis (Fig. 4c), cuticular bristles are now present and are innervated by fused HRP-positive dendrites from sensory cell clusters. By montaging optical stacks collated according to depth, we can demonstrate that HRP-positive cell clusters located distally in the epithelium of A1 and A2 project axons topologically onto their respective ventral or dorsal antennal tracts.

### Topographic and temporal organization of a sensory tract

**Fig. 5:**
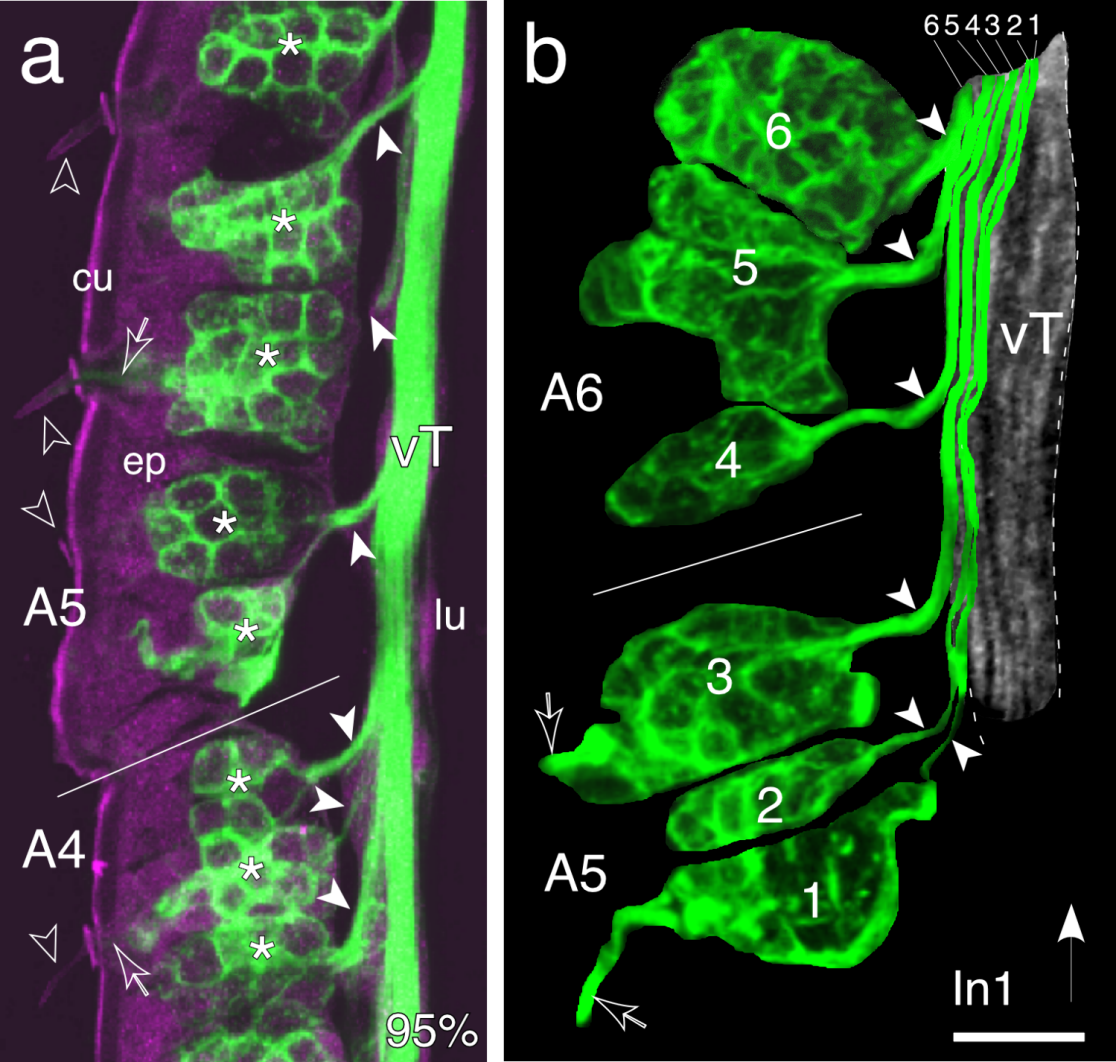
Axons from clusters of cells associated with basiconic bristles project topographically into a tract of the antennal nervous system. **a** A confocal image of a longitudinal section through the antenna in side view following immunolabeling with anti-horseradish peroxidase (α-HRP, green) at 95% of embryogenesis shows an array of sensory cell clusters (white stars) in the epithelium (ep) of segments A4 and A5 of the flagellum (white line indicates approximate border). Cuticular/epithelial autofluorescence appears magenta. Sensory dendrites (open white arrows) are seen innervating basiconic bristles (open/white arrowheads) on the cuticle (cu). Bundled axons (white arrowheads) from each cell cluster exit the epithelium and join the ventral nerve tract (vT) in the lumen (lu) in sequential order from distal to proximal (direction indicated by the white arrow in panel b). **b** Higher power confocal image of a longitudinal section through the antenna (side view) of a first instar (In1) locust following labeling against horseradish peroxidase (α-HRP, green) shows computer-based autotracings of six HRP-positive sensory cell clusters numbered sequentially from distal to proximal in the epithelium of A5 and A6 (white line is the approximate border). Autotraced axons from each cluster (white arrowheads) join the α-HRP-labeled vT (false color gray, outlined dashed white) topographically according to the location of the cell cluster in the epithelium (axon and cell cluster numbers correspond). Axons from distal clusters lie medially in the tract, and those from progressively more proximal clusters add laterally to the tract in sequence producing a topographic effect akin to tree rings. Dendrites (open white arrows) from cell clusters 1 and 3 innervating basiconic bristles in A5 are also imaged. Scale bar represents 20 µm in **a**, 10 µm in **b**

The next question was whether cell cluster location in the epithelium and hence bristle location on the cuticle are represented along with cluster age within the axon profile of a sensory tract. To realize this, we labeled cell clusters, their axon projections, together with the axonal organization of a tract, with α-HRP in longitudinally sectioned antennae. Confocal imaging of flagellar segments A4 and A5 at 95% of embryogenesis (Fig. 5a) reveals an array of sensory cell clusters in the epithelium. Sensory dendrites are seen innervating basiconic-type bristles on the cuticle, and bundled axons from each cell cluster join a nerve tract in sequential order from distal to proximal.

**Fig. 6:**
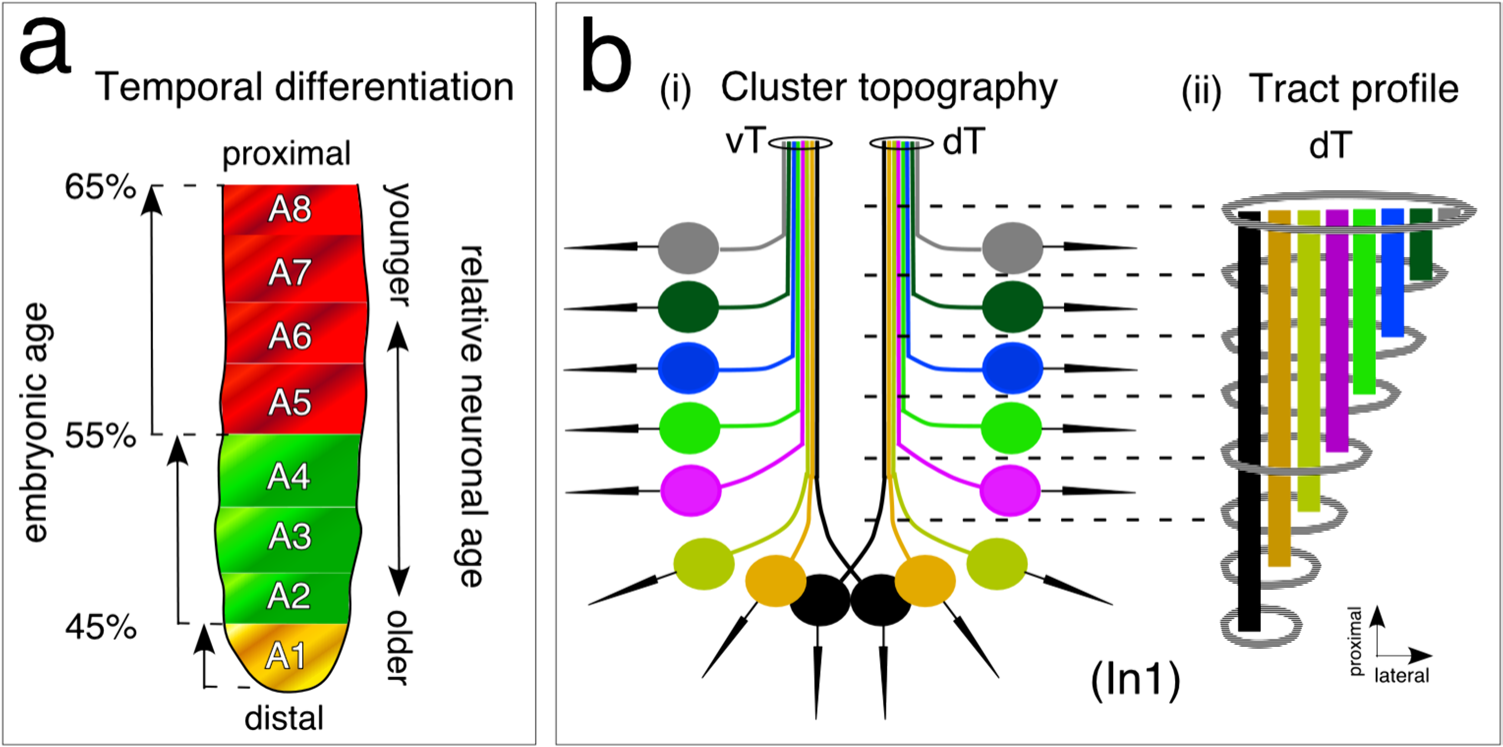
Schematics (not to scale) summarize the spatial and temporal organization of sensory cell clusters and their axonal projections in the developing antenna. **a** Temporal differentiation. Cell clusters appear in the epithelium in a distal to proximal direction providing a temporal topology to cluster organization. Cell clusters first appear in annulus A1 (yellow), then in A2–A4 (green), and finally in A5–A8. Patterns are complete in these annuli at 65% of embryogenesis. The pattern suggests that neurons from distal cell clusters are older than those in more proximal annuli. **b** (i) Cluster topography. Cell clusters (colored circles) receiving sensory input from cuticular basiconic-type bristles (black arrows) are arrayed in the epithelium of the distal antenna. Note that the depicted cluster location is representative but in sum covers the most distal six annuli. Axons from a given cluster project topographically to either a ventral (vT) or dorsal (dT) tract and join the tract commensurate with the location of the cluster along the array. (ii) Tract profile. A longitudinal sketch of the dorsal tract (dT, also applies to the vT) taken at different levels along the antenna summarizes axon topology in the tract. Clusters and axons are color matched. The tract increases in diameter as younger axons from progressively more proximal clusters join. The location of axons in the tract is stereotypic and so reflects the topography and temporal topology of cell clusters in the epithelium

To analyze axon locations within the tract, we performed computer-based autotracings of axons projecting to a tract from six representative HRP-positive sensory cell clusters. These were numbered sequentially from distal to proximal along the antenna in A5 and A6 at the first instar (In1) stage (Fig. 5b). We found that autotraced axons stereotypically add laterally to the tract as one proceeds proximally along the flagellum. Since axons join the tract strictly in order according to the location of the cell cluster in the epithelium, this results in axons from distal clusters lying more medially in the tract than those from proximal clusters, producing a topographic effect akin to tree rings (see Fig. 6).

Further, since HRP-labeled cell clusters appear developmentally from distal to proximal along the antenna (see Fig. 3), the tract possesses a temporal topology so that axons from older neurons lie medially in the tract and those from younger neurons lie laterally. If this order is maintained further downstream, it would allow spatially and temporally encoded sensory information originating from basiconic cell clusters in distal annuli A1–A6 to be transmitted unmixed from the bristle to the brain.

## Discussion

A range of studies has shown that labeled lines represent an efficient mechanism for transferring and conserving sensory information within nervous systems (Henley 2021). This principle has been particularly well elucidated in the encoding of gustatory and olfactory information in both vertebrate (Brennan and Keverne 2004; Luo and Katz 2004; Pereira and Alves 2011) and invertebrate (Masson and Mustaparta 1990; Keesey and Hansson 2021) nervous systems. In our developmental study here, we examine a sensory system in the locust *Schistocerca gregaria* involving basiconic-type sensilla (see Suppl. Figure 1; Slifer et al. 1957, 1959) implicated in olfaction, gustation, and mechanoreception (Hansson et al. 1996; Ochieng et al. 1998; Boronat-Garcia et al. 2022; Cassau et al. 2023). On hatching, these sensilla are found at fixed locations on only the six most distal segments (or annuli) of the antenna (Hansson et al. 1996; Ochieng et al. 1998; Chapman 2002) and project axons to the brain (see Hansson et al. 1996). We first identify mitotically active sense organ precursors for these sensory cell clusters (Fig. 2) and then show that their progeny appear in a distal to proximal direction along the antennal epithelium (Figs. 3, 6a). The oldest neuronal populations are therefore found in cell clusters distally near the antennal tip with progressively younger populations located more proximally. Axons from cell clusters fasciculate sequentially with a tract, adding laterally according to the location of the cell cluster along the flagellum (Figs. 5, 6b, i). We propose that cell cluster location and the associated basiconic sensilla on the cuticle of distal annuli A1–A6 are represented topographically and temporally within the axon profile of the tract itself (Fig. 6b, ii).

Our evidence for unmixed information transfer involving basiconic sensilla is currently based on data limited to the distal six annuli of the antenna where these sensilla are located and where we are able to simultaneously image their cell clusters and initial axon projections. We accept that our proposal would clearly be strengthened by being able to image axon trajectories further proximally along the antenna without simultaneously having the associated cell clusters in view. This requires differential labeling of cell clusters and axons, for example via genetic labeling (Cardona et al. 2010), a technology not yet available to us in the locust.

### Developmental aspects

At the end of embryogenesis, the locust antenna comprises 11 articulations or meristal annuli (Chapman 2002), and during subsequent development, the more proximal annuli subdivide in a fixed manner to generate the adult complement of 24 annuli, whereas the most distal annuli do not subdivide (Chapman 2002) so their identity must already be established earlier during embryogenesis. Our study involving basiconic-type sensilla located distally on the locust antenna shows that both time (the temporal pattern of cell cluster appearance) and space (location along the antenna) are represented in the axon profiles of nerve tracts projecting to the brain (Fig. 6). From a spatial perspective, this anatomical organization may subserve the transfer of behaviorally relevant sensory information via odorant binding proteins in basiconic sensilla that have also been shown to be expressed topographically in the antenna of the desert locust (Jiang et al. 2018). Developmentally, SNMP1 and SNMP2 sensory neuron membrane proteins belonging to the CD36 family of lipid receptors and transporters and expressed in locust basiconic sensilla are already distributed topographically in the antenna by the first instar stage and must therefore have developed embryonically, and this pattern is subsequently retained to maturity (Cassau et al. 2023).

A similar developmental pattern is also present in another orthopteroid insect—the cockroach—where meristal annuli are progressively added to the base of the flagellum during postembryonic development so that more sensilla form proximally as nymphal development progresses (Watanabe et al. 2018). As the axons of the pheromone-sensing sensilla project to different layers in the macroglomerulus of the antennal lobe, the spatial location and date of birth of each pheromone-sensing neuron are mapped to this region. Since antennal lobe responses in the cockroach have been shown to vary depending on the spatial location of the olfactory sensilla along the length of the antenna, these represent a physiological correlate of brain neuroarchitecture (Paoli et al. 2020).

### Topographic projections and brain maps

The efficacy of point-to-point information transfer is maximized if both source and target possess a matching topography as in the barrel cortex of rodents, where somatosensory cortical organization mirrors that of the whisker follicles on the snout (Bray 2015; Petersen 2017), or olfactory pathways from the antennae and palps to the olfactory lobe in insects (Masson and Mustaparta 1990; Keesey and Hansson 2021). A spatial map of sensillum subtypes on the antenna and palps has also been reported in *Drosophila* (Keesey et al. 2022) and matches a highly ordered spatial pattern of odorant receptor expression (Vosshall et al. 1999). Combined, these factors translate into an odorant map in the antennal lobe (Fishilevich and Vosshall 2005) comparable to the functional brain maps based on peripheral mechanosensory input (Patella and Wilson 2018) as in the locust (Hansson et al. 1996) and cockroach (Nishino et al. 2015) where terminals from various odorant-sensitive afferents are distributed discretely in the olfactory lobe, paralleling the topographic organization of sensory afferents from Johnston’s organ in *Drosophila* (Kamikouchi et al. 2006), ants (Grob et al. 2021), and the honeybee brain (Ai et al. 2007; Brockmann and Robinson 2007).

Studies of labeled lines in neuronal systems have generally followed information transfer running from the periphery to central processing centers. In the visual system of *Drosophila*, for example, the axon tracts of neurons receiving input from different fields of the anterior optic tubercle are spatially segregated and terminated in different areas of the bulb (Kandimalla et al. 2023). Neurons downstream in these different bulb regions then transmit the information to different subfields in the ellipsoid body, so that a topographic map is maintained within the anterior visual pathway. In an alternative approach, Cardona et al. (2010) reverse-engineered labeled lines by applying an algorithm that allows neuronal lineages (clones) in the *Drosophila* brain to be reconstructed based on a sequence analysis of their axon trajectories. This approach is species independent and is consistent with earlier findings involving a clonal analysis of mushroom body neuroarchitecture in *Drosophila* (Ito et al. 1997, 2013) and central complex neuroarchitecture in both *Drosophila* (Young and Armstrong 2010) and the locust (Williams and Boyan 2008; Boyan et al. 2015, 2017). In the developing central complex of both the locust (Williams et al. 2005; Williams and Boyan 2008) and *Drosophila* (Young and Armstrong 2010) brain, four clones of neurons whose axons project via discrete tracts into the central complex have been shown to be internally organized according to age. Constituent neurons direct axons topologically according to age into the respective tract, thus generating a clonally based modular system of fiber tracts whose topology translates into columnar projections consistent with the neuroarchitecture of the adult brain (Williams 1975; Williams et al. 2005; Strausfeld 2009; Young and Armstrong 2010; Boyan and Liu 2016). Comparisons of topographic decussation in the central complex across a range of arthropods have allowed equivalent neuropilar structures to be identified and so provide insights into phylogenetic relationships among the Panarthropoda (Strausfeld 2012; Boyan et al. 2015).

### Ecological aspects

Our study focusses on the projection patterns of basiconic-type sensilla that constitute a subset of the sensilla population present on the most distal segments of the locust antenna at the first nymphal stage. Previous studies have shown that the distribution of sensilla on more proximal segments of acridid antennae changes during postembryonic development (Chapman and Greenwood 1986; Chapman 2002), is influenced by environmental effects (Chapman and Lee 1991), and varies according to diet (Chen et al. 2003; Nakano et al. 2022), and whether the locusts are in solitarious or gregarious phases (Greenwood and Chapman 1984; Ochieng and Hansson 1999). Expression of odorant receptors also varies by sex (Boronat-Garcia et al. 2022), and this may account for sex-based differences in the physiological responses of olfactory neurons (Ochieng and Hansson 1999). Interestingly, although ecologically induced changes to functional ligands in the olfactory system of *Drosophila* may not affect all olfactory sensory neurons equally, the underlying neuronal wiring appears to be conserved despite ligand spectrum shifts (Keesey et al. 2022). Neural circuitry involving labeled lines therefore appears to represent a stable element in an otherwise molecularly dynamic sensory system. The challenge is to now incorporate these various ecological, developmental, and molecular aspects into a neuronal framework that provides a substrate for adaptive behavior.

### Supplementary information

### Supplementary Information

Below is the link to the electronic supplementary material.

Supplementary file1 (TIF 914 KB)

Supplementary file2 (TIF 833 KB)

## Data Availability

Core data supporting this study are available on request from Dr. E.E. Ehrhardt, AG Ito, Institute of Zoology, Universität Köln, Zülpicher Str 47b, 50,674, Cologne, Germany.

## Declarations

### Third-party permissions

Permission has been obtained from the copyright holder to reproduce Fig. 1 from Slifer et al. 1957 in modified form. This permission can be provided on request.
