# Supplementary figures and images for: From bristle to brain: embryonic development of topographic projections from basiconic sensilla in the antennal nervous system of the locust Schistocerca gregaria
Source: Dev Genes Evol. 2024 May 1;234(1):33–44. doi: 10.1007/s00427-024-00716-2 (PMC11226553; doi:10.1007/s00427-024-00716-2)

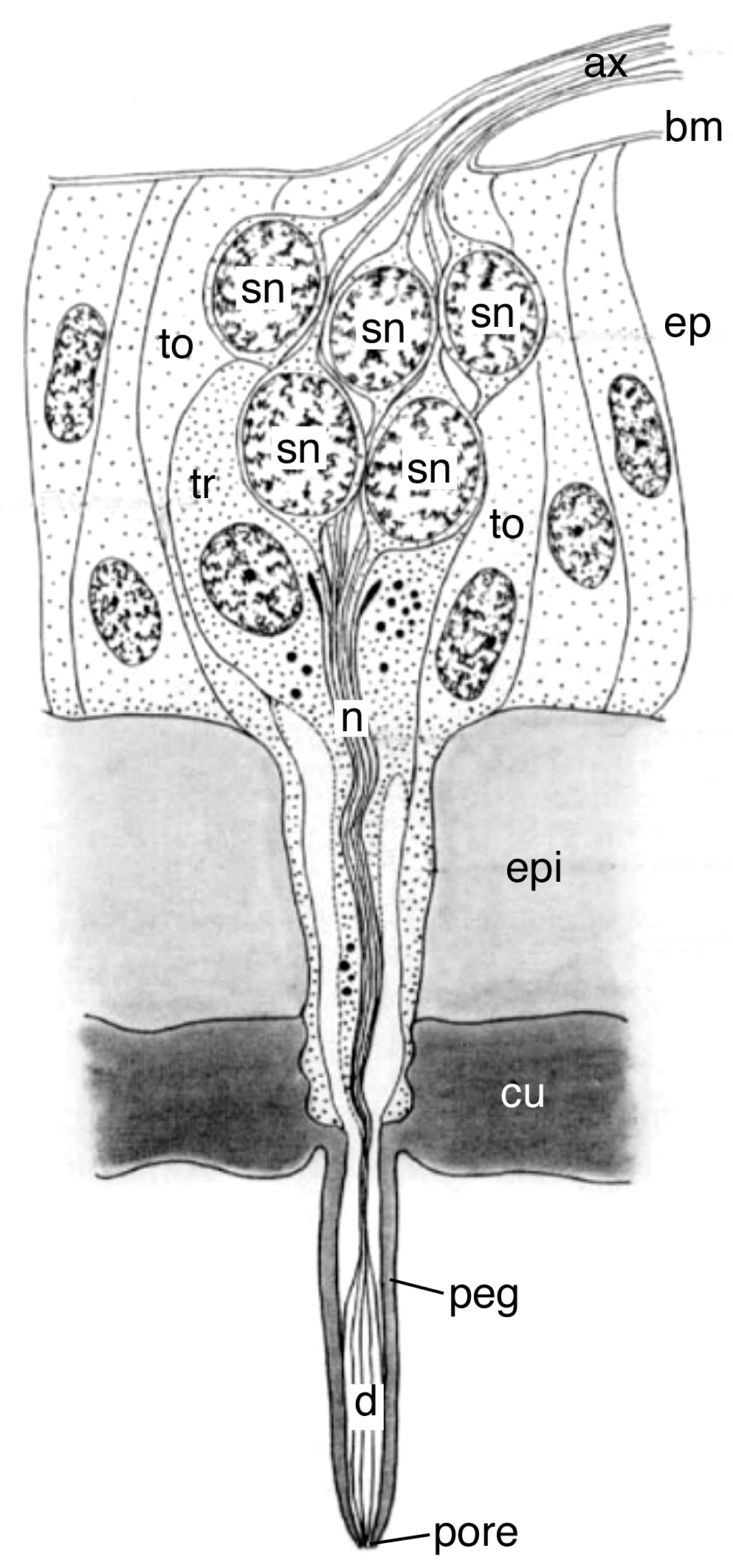

Supplement: Supplementary file 1 — Supplementary file1 (TIF 914 KB) [file 427_2024_716_MOESM1_ESM.tif]

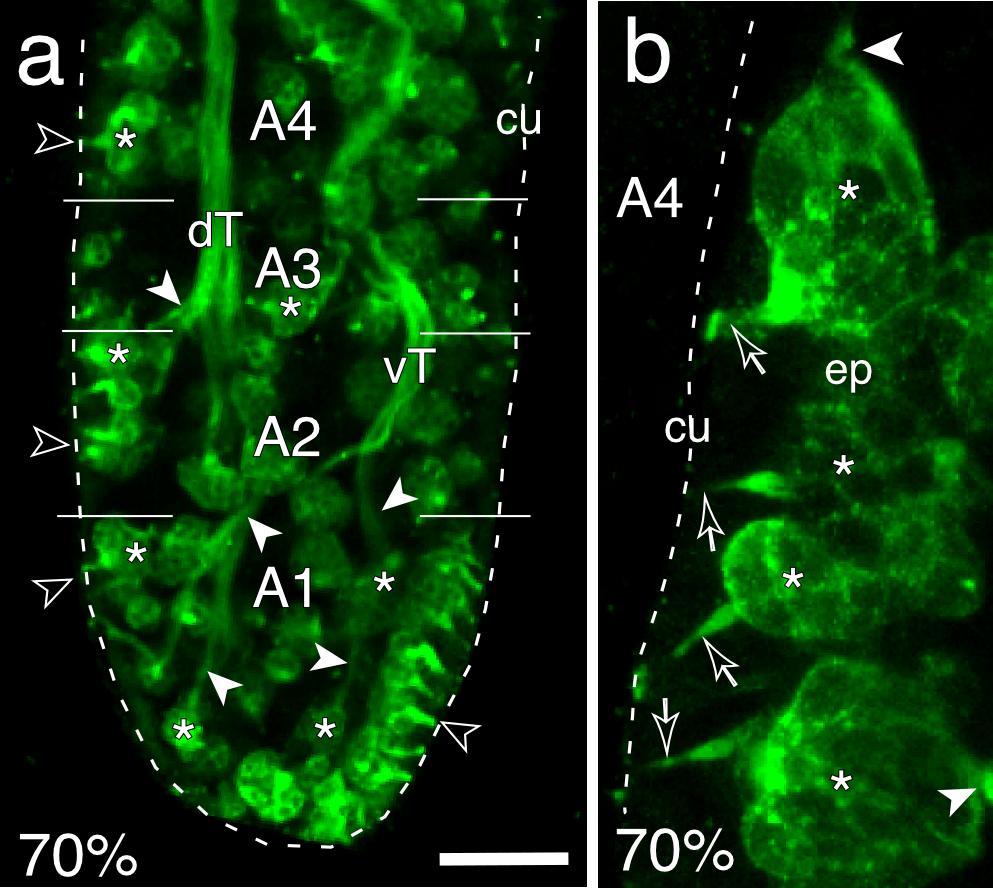

Supplement: Supplementary file 2 — Supplementary file2 (TIF 833 KB) [file 427_2024_716_MOESM2_ESM.tif]
